# Analysis of Ginsenoside Content (*Panax ginseng*) from Different Regions

**DOI:** 10.3390/molecules24193491

**Published:** 2019-09-26

**Authors:** Wei Chen, Prabhu Balan, David G Popovich

**Affiliations:** 1 School of Food and Advanced Technology, Massey University, Palmerston North 4442, New Zealand; W.Chen2@massey.ac.nz; 2 Riddet Institute, Massey University, Palmerston North 4442, New Zealand; P.Balan@massey.ac.nz; 3 Alpha-Massey Natural Nutraceutical Research Centre, Massey University, Palmerston North 4442, New Zealand

**Keywords:** *Panax ginseng*, ginsenosides, New Zealand, China, Korea

## Abstract

Recently *Panax ginseng* has been grown as a secondary crop under a pine tree canopy in New Zealand (NZ). The aim of the study is to compare the average content of ginsenosides from NZ-grown ginseng and its original native locations (China and Korea) grown ginseng. Ten batches of NZ-grown ginseng were extracted using 70% methanol and analyzed using LC-MS/MS. The average content of ginsenosides from China and Korea grown ginseng were obtained by collecting data from 30 and 17 publications featuring China and Korea grown ginseng, respectively. The average content of total ginsenosides in NZ-grown ginseng was 40.06 ± 3.21 mg/g (n = 14), which showed significantly (*p* < 0.05) higher concentration than that of China grown ginseng (16.48 ± 1.24 mg/g, n = 113) and Korea grown ginseng (21.05 ± 1.57 mg/g, n = 106). For the individual ginsenosides, except for the ginsenosides Rb2, Rc, and Rd, ginsenosides Rb1, Re, Rf, and Rg1 from NZ-grown ginseng were 2.22, 2.91, 1.65, and 1.27 times higher than that of ginseng grown in China, respectively. Ginsenosides Re and Rg1 in NZ-grown ginseng were also 2.14 and 1.63 times higher than ginseng grown in Korea. From the accumulation of ginsenosides, New Zealand volcanic pumice soil may be more suitable for ginseng growth than its place of origin.

## 1. Introduction

*Panax ginseng*, as one of the most important medicinal plant, root was used for first aid, health care, and the treatment of coma, gastrointestinal disease, and cardiovascular disease in ancient China [1]. Pharmacological studies found ginseng possesses diverse bioactive effects, such as anti-aging, anti-stress, anti-tumor, anti-inflammatory, and anti-diabetes [2]. It has a beneficial impact on brain function, liver function, immune function, and sexual function [3]. Since the use of traditional Chinese herbs for medicinal and dietary purposes is becoming increasingly popular in Western countries, ginseng is becoming one of the best-selling herbs in the world [4].

**Figure 1:**
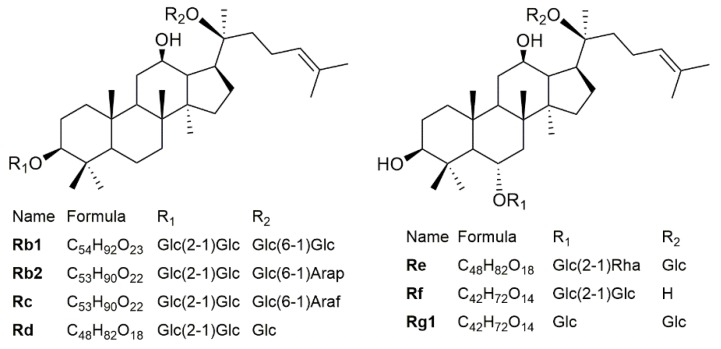
The chemical structures of main ginsenosides. Glc, Arap, Araf, and Rha refer to β-D-glucopyranosyl, α-L-arabinopyranosyl, α-L-arabinofuranosyl, and α-L-rhamnopranosyl, respectively.

Ginsenosides are known as the key active ingredient of ginseng [2]. The diversity of ginseng efficacy is related to the structural variability of ginsenosides, also known as triterpenoid saponins [5]. More than a hundred ginsenosides have been reported in *Panax ginseng* [6]. Among them, ginsenosides Rb1, Rb2, Rc, Rd, Re, Rg1 (chemical structures are shown in Figure 1) are the most abundant and are usually regarded as the main ginsenosides. These six ginsenosides are normally used to evaluate ginsenoside abundance and for quality control. The accumulation of ginsenosides in ginseng is variable and can be influenced by the surrounding environment, including soil fertility, temperature, light, and humidity [7]. Even in the same area, the type and amount of ginsenosides are dependent on ginseng age [8], harvest time [9], and storage process [10]. Since ginseng’s bioactive properties depend largely on the contents and types of ginsenosides, accurately knowing the amount of ginsenosides is not only important for the pharmacological evaluation of ginseng products, but also for assessing the quality of ginseng from different regions.

With the increasing popularity of ginseng around the world, ginseng is also cultivated outside its place of origin (Northeast China, and Korea). Recently ginseng has been grown in New Zealand (NZ). NZ has a unique geographical environment, such as the volcanic pumice soil, high-intensity UV rays, and open-wild environment. NZ-grown ginseng showed abundant ginsenosides in the underground parts (roots, rootlets) and above ground parts (stem and leaf) based on our previous study [11]. To compare the average amount of ginsenosides between NZ-grown ginseng and ginseng grown in China and Korea, we analyzed the amount of ginsenosides in different batches of ginseng grown in NZ under a pine forest canopy and compared it to published data. Thus, this paper not only provides the information about the differences of ginsenoside content in ginseng from different places of origin, but it also provides a reference data for the saponin content from ginseng produced in different areas. To our knowledge, this is the first article focusing on ginsenoside content in different regions.

## 2. Results and Discussion

### 2.1. The Ginsenoside Contents of NZ-Grown Ginseng

In this study, the amount of 21 ginsenosides in 10 batches of ginseng samples were analyzed using LC-MS/MS described in our previous method [11]. Briefly, thirteen ginsenosides (Rb1, Rb2, Rb3, Rc, Rd, Re, Rf, Rg1, Rg2, Rg3, Rh1, Rh2, and F2) were accurately quantified by their own linear regression equations of standard curves, and some ginsenosides without reference standards, such as ginsenosides m-Rb1 (m = malonyl), m-Rb2, m-Rb3, m-Rc, m-Rd, m-Re, and m-Rg1, were relatively quantified by the regression equations of their corresponding neutral ginsenosides. As shown in Table 1, apart from the individual ginsenoside content, the total ginsenoside amount, the ratio of protopanaxadiol (PPD)-type to protopanaxatriol (PPT)-type (PPD/PPT) and the ratio of neutral ginsenoside to malonyl ginsenoside (G/m-G) are also described in this section.

Among those samples, the ginsenoside concentrations were varied. The highest amount (102.69 ± 0.91 mg/g) and the lowest amount (38.72 ± 0.15 mg/g) were from the third batch and fifth batch of samples, respectively. Ginsenosides Rg1, Rb1, m-Rb1, and Re are the four major ginsenosides in most samples except the fourth and fifth batches of samples, while in the fourth and fifth batches of samples, the major ginsenosides give way to the ginsenosides m-Rb1, m-Rb2, m-Rc, and Rg1 based on their concentrations. The high amount of m-Rb1, m-Rb2, and m-Rb3 lead to the higher ratios of PPD/PPT and lower ratios of G/m-G in sample four and five. Both ratios are approximately two from other samples.

Usually, ginseng refers to the whole ginseng root including the main root body, root hair, and rhizome. In our previous study, we analyzed ginsenosides from different parts including main root, fine root, rhizome, stem, and leaf [11], it was found that the underground parts, including main root, fine root, and rhizome, have very similar ginsenoside composition. In this study ginsenosides were determined from the whole underground part. There is another study reporting ginsenosides analysis for NZ-grown ginseng [12], which only detected seven main ginsenosides in four batches of ginseng samples. In order to compare with China and Korea grown ginseng, the data from Table 1 and Follett’s publication [12] are used to calculate the average content of ginsenosides of NZ-grown ginseng.

### 2.2. The Average Content of Ginsenosides in Different Regions

**Figure 2:**
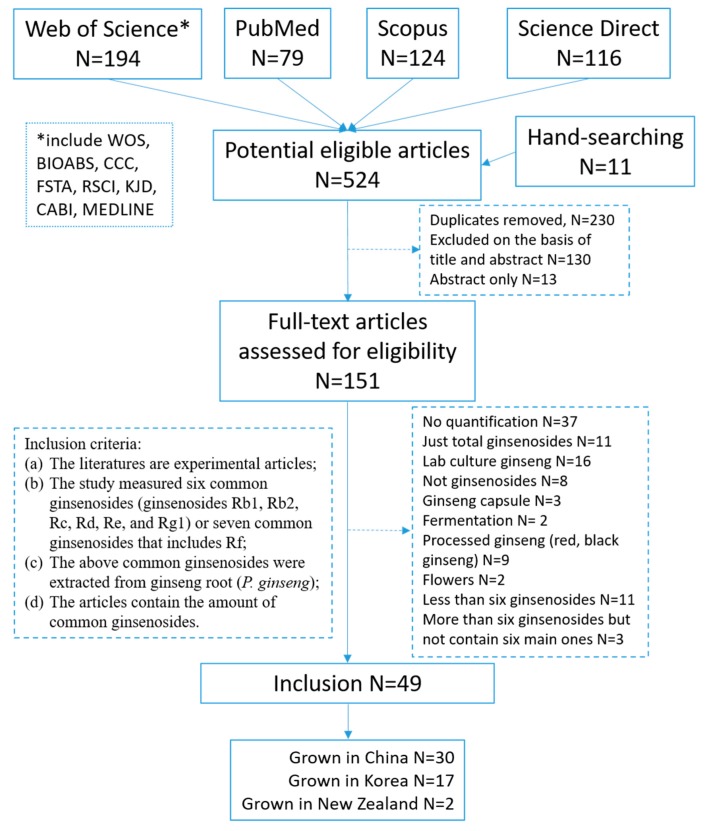
Flow diagram of study search and inclusion criteria.

In order to obtain the average content of ginsenosides from China grown ginseng and Korea grown ginseng, we searched the scientific literature for ginsenosides analysis from ginseng grown in China and Korea. A total of 524 articles were located, and 151 potentially relevant studies were selected to full-text review after removing the duplicates (N = 230), abstract only (N = 13) and screening on the basis of title and abstract according to the inclusion criteria. After exclusion of 102 articles for the reasons given in Figure 2, we included 49 articles (article lists are supplied in the Supplementary Materials). Among these articles, 30 articles were about ginsenosides analysis for China grown ginseng, 17 for Korea grown ginseng, and two for NZ-grown ginseng.

After extracting the data from the inclusion articles, the amount of ginsenosides from 113 batches of China grown ginseng roots aged from three to 30-years old was used to calculate the average content of ginsenosides of China grown ginseng. A total of 106 batches of Korea grown ginseng roots with different ages (one to 10-years old) were analyzed from 17 publications. For NZ-grown ginseng, there were 14 batches of samples tests, including ten batches of ginseng roots in this study and four from the literature.

**Figure 3:**
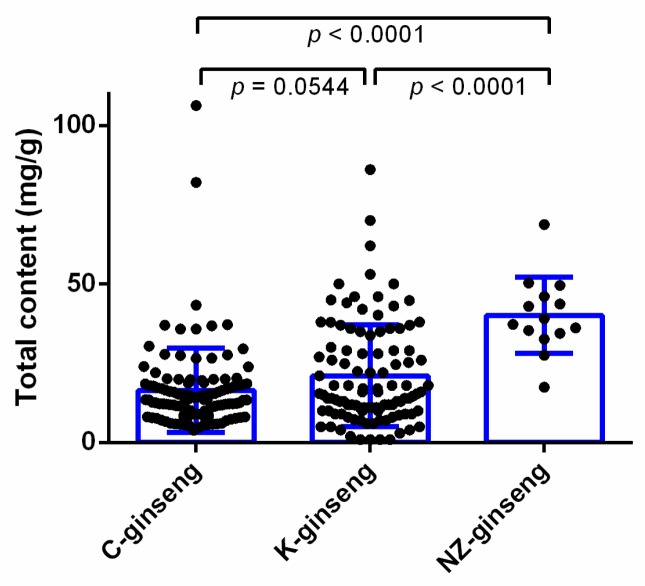
The average content of total ginsenosides in the ginseng roots grown in three different countries. Data were analyzed by one-way ANOVA using Graph pad prism 6 software and expressed as mean ± SE. Differences were considered significant if *p* < 0.05. Each dot represents one batch of ginseng sample test datum. C, K, and NZ-ginseng refers to China (n = 113), Korea (n = 106), and New Zealand (n = 14) grown ginseng, respectively.

As shown in Figure 3, the average content of total ginsenosides from NZ-grown ginseng (40.06 ± 3.21 mg/g, n = 14) is significantly (*p* < 0.05) 1.4 times higher than that of China grown ginseng (16.48 ± 1.24 mg/g, n = 113) and 90% higher compared with that of Korea grown ginseng (21.05 ± 1.57 mg/g, n = 106). There is no significant difference in the average content of total ginsenosides for ginseng grown in China and Korea.

**Figure 4:**
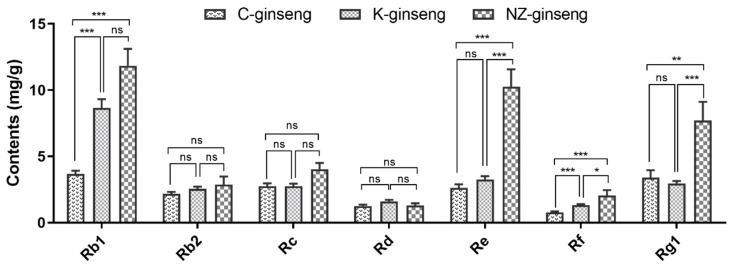
The average content of individual ginsenosides in the ginseng roots grown in three different countries. Data were analyzed by one-way ANOVA using Graph pad prism 6 software and expressed as mean ± SE. * *p* < 0.05, ** *p* < 0.01, *** *p* < 0.001. Differences were considered significant if *p* < 0.05. C, K, and NZ-ginseng refers to China (n = 113), Korea (n = 106), and New Zealand (n = 14) grown ginseng, respectively.

The average contents of seven individual ginsenosides are shown in Figure 4, the average concentrations of ginsenoside Rb1, Re, Rf, and Rg1 from NZ-grown ginseng are significantly (*p* < 0.05) 2.22, 2.91, 1.65, and 1.27 times higher than that of China grown ginseng, respectively. The ginsenosides Re, Rf, and Rg1 of New Zealand ginseng are 3.14, 1.55, and 2.63 times that of Korean ginseng, respectively. The average content of PPD type ginsenosides Rb2, Rc, and Rd are not significantly different among the three growing regions of ginseng.

**Figure 5:**
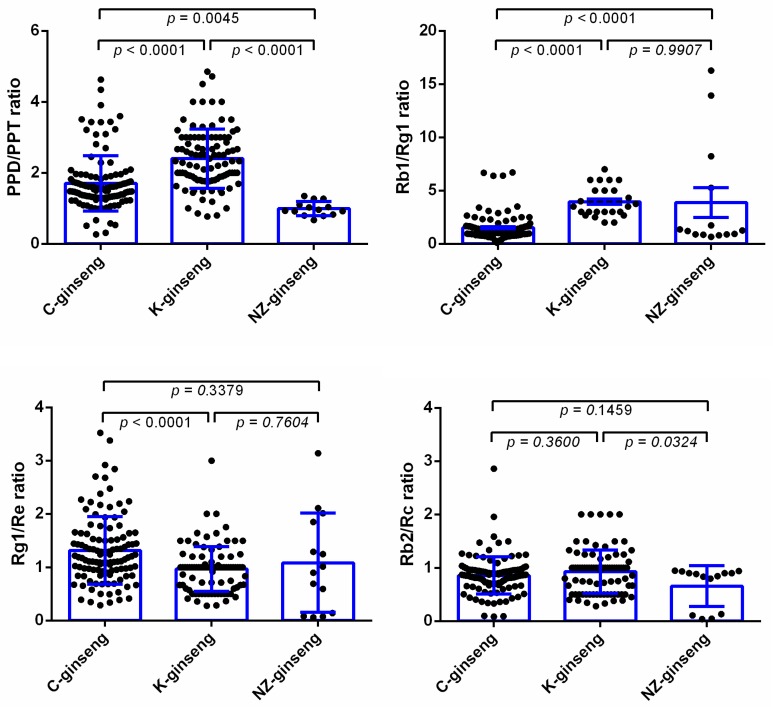
The PPD/PPT, Rb1/Rg1, Rg1/Re, and Rb1/Rc ratios of ginsenosides in the ginseng roots grown in three different countries. Data were analyzed by one-way ANOVA using Graph pad prism 6 software and expressed as mean ± SE. Differences were considered significant if *p* < 0.05. Each dot represents one batch of ginseng sample test datum. C, K, and NZ-ginseng refers to China (n = 113), Korea (n = 106), and New Zealand (n = 14) grown ginseng, respectively.

It is reported that the ratios of PPD to PPT and Rb1 to Rg1 are less than 2.0 and less than 5.0 in *Panax ginseng*, respectively [13]. Thus, we also calculated these ratios of ginsenosides from three countries (Figure 5). The PPD/PPT ratios of all the NZ-grown ginsengs are less than 2.0, although the average ratio of PPD/PPT from China grown ginseng is less than 2.0, many of the ratios are more than 2.0. While, most values of PPD/PPT from Korea grown ginseng are more than 2.0, leading to the average with >2.0. Although the average ratios of Rb1/Rg1 are less than 5.0 among three countries, some ginseng samples still have Rb1/Rg1 > 5.0. In addition, the ratios of Rg1 to Re is more than 1.0, and the ratio of Rb2 to Rc is more than 0.4 in *Panax ginseng* [14]. From the average level, the average ratios of Rg1/Re and Rb2/Rc are more than 1.0 and 0.4, respectively, among the three regions’ ginseng samples. However, neither the value of Rg1/Re stays >1.0 all the time, nor the value of Rb2/Rc shows >0.4 in all samples.

Since the contents of PPT type ginsenosides (Re, Rf and Rg1) in NZ-grown ginseng are significantly higher than that of Chinese ginseng and Korean ginseng, besides Rb1, the concentrations of other PPD type ginsenosides (Rb1, Rc and Rd) have no significant difference among three regions’ ginseng, which lead to the lower PPD/PPT ratio in NZ-grown ginseng.

As the two most important sources of ginseng production, the total production by China and Korea is 72,229 tons, which was approximately 90.2% of the world ginseng production [15]. Many studies have reported the ginsenoside concentrations from China and Korea. However, they mainly focused on one batch or limited batches of sample analysis [16,17,18]. There are few studies concerning the comparison of ginsenosides between ginseng grown in different countries. We did not know if they have some differences in the ginsenosides content between China and Korea grown ginseng. In this study, the average content of ginsenosides from hundreds of batches of ginseng samples represent the ginsenoside levels of ginseng grown in different regions. We can see there is neither a significant difference in the content of the total ginsenosides between China grown ginseng and Korea grown ginseng, nor remarkably differences in the individual ginsenoside contents of Rb2, Rc, Rd, Re, Rf, and Rg1 in both countries. This may be due to the geographical proximity of Northeast China and South Korea, and they have a similar growing environment for ginseng.

However, NZ-grown ginseng is grown in the Southern Hemisphere, apart from the similar growth conditions including the cold winter, temperate summer, and weakly acidic soil to that ginseng’s natural habitat in Northeast of China and Korea [19], there are some different growing characteristics in New Zealand, including the volcanic pumice soil and high intensity light radiation. A recent study showed that photosynthetically active radiation, soil, and water potentially had a great impact on ginsenoside accumulation in ginseng roots [20]. The volcanic pumice soil can provide an excellent environment for root growth because of its unique properties such as dark soil color, unique consistency, low bulk density, difficult clay dispersion, and high water holding capacity [21]. Additionally, during the Southern Hemisphere summer, NZ receives, on average, 7% more radiation compared to a given latitude in the Northern Hemisphere summer [22]. It was reported that the total ginsenoside content increased significantly until light transmission rate increased by 20%, but the PPT type ginsenosides increased larger than PPD type ginsenoside, leading to the ratio of PPD/PPT eventually decreasing [23]. So to some extent, we have reason to believe that NZ’s unique geographical environment encourages elevated ginsenosides (especially PPT type ginsenosides) content compared to ginseng grown in the Northern Hemisphere with similar latitudes. On the other hand, we need to note the limitation of this study, there is only 14 batches of NZ-grown ginseng sample data, a bit smaller compared to hundreds of batches of sample data of Chinese ginseng and Korean ginseng.

## 3. Materials and Methods

### 3.1. Analysis of Ginsenosides Content of NZ Grown Ginseng

#### 3.1.1. Ginseng Samples

All the NZ-grown ginseng samples were collected from pine forest around Taupo and Rotorua (Table 2). The samples were rinsed with water, lyophilized at −68 °C and powered.

#### 3.1.2. Standard Samples, Chemicals and Regents

Thirteen reference standards of ginsenosides Rb1, Rb2, Rb3, Rc, Rd, Re, Rf, Rg1, Rg2, Rg3, Rh1, Rh2, and F2 were purchased from Star Ocean Ginseng Ltd. (Suzhou, Jiangsu, China). The purities of all reference standards were above 98.0%. HPLC-grade methanol (MeOH) and formic acid (HCOOH) were purchased from Fisher Chemical (Pittsburg, PA, USA). LC-MS-grade acetonitrile (MeCN) and water were obtained from Merck (Phillipsburg, NJ, USA). Water (for extraction) was obtained from a Milli-Q Ultra-pure water system (Millipore, Billerica, MA, USA). Other reagents used in this study were of analytical grade.

#### 3.1.3. Sample Preparation and HPLC-QTOF-MS/MS Analysis

Ginsenosides were extracted three times from ginseng samples using a Q700 sonicator (Qsonica, Melville, NY, USA) and analyzed by an Agilent 1290 liquid chromatograph coupled with quadrupole time-of-flight tandem mass spectrometry (Agilent, MA, USA) according to our previous methods [11]. Briefly, 0.7 g dried ginseng root powder was mixed with 10 mL 70% (*v*/*v*) aqueous MeOH and extracted at 20 kHz for 10 min at no more than 40 °C. (The extraction was carried out for five cycles, each cycle contained 2 min ultrasonically extraction at 15% amplitude and 1 min for cooling between extraction). The supernatant was collected after centrifugation at 4000 rpm for 10 min and the sediment was extracted twice more. The three extracts were mixed together and filtered through a 0.22-μm filter before LC/MS analysis.

Thirteen reference standards of ginsenosides Rb1 (0.769 mg/mL), Rb2 (0.846 mg/mL), Rb3 (0.629 mg/mL), Rc (1.077 mg/mL), Rd (0.692 mg/mL), Re (0.923 mg/mL), Rf (1.462 mg/mL), F2 (0.692 mg/mL), Rg1 (1.154 mg/mL), Rg2 (0.615 mg/mL), Rg3 (1.154 mg/mL), Rh1 (1.000 mg/mL), and Rh2 (1.077 mg/mL) were dissolved in 70% MeOH and then mixed and diluted with 70% MeOH to obtain a series of mixture standard solutions of different concentrations. The solutions were filtered through a 0.22- μm syringe filter before LC/MS analysis.

An Agilent 1290 liquid chromatograph (Agilent, MA, USA) equipped with an online degasser, an auto-sampler, a quaternary pump, and a heated column compartment, and an Agilent 6530 Quadrupole Time of Flight Mass Spectrometer (Agilent, MA, USA) equipped with an electrospray ionization source were used for LC/MS analysis. The separation was based on a Zorbax Extend-C18 (2.1 × 100 mm, 3.5 μm) column (Agilent, USA) at a temperature of 33 °C. The binary gradient elution solvent consisted of water (A) and acetonitrile (B) (Both A and B containing 0.1% formic acid). The gradient elution program was as follows: 0–4 min, 80% A; 4–10 min, 80%–70% A; 10–25 min, 70%–67.5% A; 25–27 min, 67.5%–40% A; 27–39 min, 40%–5% A; 39–40 min, 5% A; 40–40.5 min, 5%–80% A. The flow rate was changed with the gradient: 0–27 min, 0.2 mL/min; 27–40.5 min, 0.25 mL/min. The injected volume was 1 μL. The mass spectrometer data were collected from *m*/*z* 100–2200 in negative ion model and nitrogen (>99.998%) was used for nebulizer gas and curtain gas. The gas temperature and flow rate were 350 °C and 10.0 L/min, respectively. The pressure of nebulizer was 37 psi. The voltages of capillary, fragmentor, and skimmer were 3500 V, 220 V, and 65 V, respectively. The reference masses in negative ion mode were at *m*/*z* 121.0509 and 922.0098. The acquisition rates were 4 spectra/s for MS and 1 spectrum/s for MS/MS. Mass data were analyzed with Agilent MassHunter Workstation software (version B.06.00; Agilent Technologies, Santa Clara, CA, USA).

### 3.2. Analysis of Ginsenosides Content from China and Korea Grown Ginseng

Ginsenosides content of China and Korea grown ginseng obtained from publications based on the following search criteria.

#### 3.2.1. Search Strategy

The following search terms were applied to search the following electronic databases from their inception to April 2019.

The following search terms were used in Web of Science: TITLE: (Content* OR level* OR amount* OR quantif* OR determinat* OR investigat* OR analy*) AND TOPIC: (Ginsenoside*) AND TITLE: (“Panax ginseng” OR “Korean ginseng”). Refined by: LANGUAGES: (ENGLISH OR CHINESE) AND DOCUMENT TYPES: (ARTICLE). Timespan: All years. Databases: WOS, BIOABS, CABI, CCC, FSTA, KJD, MEDLINE, RSCI, SCIELO. Search language = Auto.

The following search terms were used in the PubMed database: (Ginsenoside [Title] AND content [Title]) OR (ginsenosides [Title] AND content [Title]) OR (ginsenoside [Title] AND contents [Title]) OR (ginsenosides [Title] AND contents [Title]) OR (ginsenosides [Title] AND investigation [Title]) OR (ginsenosides [Title] AND quantification [Title]) OR (ginsenosides [Title] AND determination [Title]) NOT rat [Title]).

The following search terms were used in Science Direct: Find articles with these terms “content of ginsenosides”; Title, abstract, keywords: “ginsenoside, Panax ginseng”; Article type: Research articles.

The following search terms were used in Scopus: TITLE-ABS-KEY (ginsenosides) AND (LIMIT-TO (ACCESSTYPE (OA))) AND (LIMIT-TO (SUBJAREA, “PHAR”) OR LIMIT-TO (SUBJAREA, “CHEM”) OR LIMIT-TO (SUBJAREA, “AGRI”)) AND (LIMIT-TO (EXACTKEY- WORD, “Article”) OR LIMIT-TO (EXACTKEYWORD, “Ginsenoside”) OR LIMIT-TO (EXACTKEYWORD, “Ginsenosides”) OR LIMIT-TO (EXACTKEYWORD, “Ginseng”)) AND (LIMIT-TO (LANGUAGE, “English”) OR LIMIT-TO (LANGUAGE, “Chinese”)) AND (LIMIT-TO (SRCTYPE, “j”)) AND (EXCLUDE (EXACTKEYWORD, “Animals”) OR EXCLUDE (EXACTKEYWORD, “Animal Experiment ”)) AND (EXCLUDE (EXACTKEYWORD, “Human”)).

#### 3.2.2. Inclusion Criteria

The following inclusion criteria were defined: (a) The literature are experimental articles; (b) the study measured six common ginsenosides (ginsenosides Rb1, Rb2, Rc, Rd, Re, and Rg1) or seven common ginsenosides that includes Rf; (c) the above common ginsenosides are extracted from ginseng root (*Panax ginseng*); (d) the articles contain the amount of common ginsenosides.

#### 3.2.3. Exclusion Criteria

The following exclusion criteria were defined: (a) Excludes articles about ginsenoside contents in the animal or human tissue samples, such as plasma, urine, and feces, etc.; (b) excludes articles that the materials contain other ingredients, such as Chinese herbs formula, commercial ginseng products (c) excludes articles that ginseng materials did not grow in the forest or farmland, such as cultured laboratory samples.

#### 3.2.4. Data Extraction

Investigators reviewed the titles, abstracts, and full text of the resulting articles for inclusion. Data were extracted from each publication meeting the inclusion criteria. The values of ginsenosides (Rb1, Rb2, Rc, Rd, Re, Rf, and Rg1) content (mean) were collected based on grown locations (China, Korea, and NZ). When ginsenosides content were reported using bar charts in the literature, the estimated values were collected using ruler assistance.

### 3.3. Data Analysis

The level of ginsenosides of NZ-grown ginseng was expressed as mean ± SD, and the average contents of ginsenosides of different regions from literature were calculated and expressed as mean ± SE. One-way ANOVA was used to analyze the difference among groups. A *p*-value < 0.05 was considered to be statistically significant.

## 4. Conclusions

This is the first article focusing on ginsenoside content in different producing areas. In this study, the average content of ginsenosides from three regions (China, Korea, and NZ) are described and compared. There is no significant difference in the average content of total ginsenosides between China grown ginseng and Korea grown ginseng. NZ-grown ginseng has significantly higher average content of total ginsenosides than that of above two regions, especially higher concentrations of PPT type ginsenosides Re, Rf, and Rg1.

## Figures and Tables

**Table 1:** The content (mg/g) of ginsenosides in 10 batches of New Zealand (NZ)-grown ginseng samples (mean ± SD).

Compound	Ginseng Samples									
	1	2	3	4	5	6	7	8	9	10
Rg1	9.25 ± 0.15	8.49 ± 0.00	14.56 ± 0.17	6.93 ± 0.00	3.22 ± 0.02	14.01 ± 0.06	15.54 ± 0.04	8.17 ± 0.30	12.79 ± 0.03	9.16 ± 0.10
Rf	2.41 ± 0.09	2.79 ± 0.05	4.01 ± 0.09	2.01 ± 0.01	1.07 ± 0.01	3.13 ± 0.00	4.13 ± 0.08	2.76 ± 0.01	3.05 ± 0.08	3.25 ± 0.02
Re	4.38 ± 0.02	12.27 ± 0.05	11.67 ± 0.17	5.37 ± 0.00	5.41 ± 0.04	4.47 ± 0.03	8.40 ± 0.10	8.04 ± 0.10	6.36 ± 0.11	10.18 ± 0.38
Rg2	0.41 ± 0.00	0.89 ± 0.00	0.89 ± 0.02	0.12 ± 0.00	0.40 ± 0.00	0.33 ± 0.00	0.69 ± 0.01	0.67 ± 0.00	0.46 ± 0.01	0.87 ± 0.01
Rh1	0.02 ± 0.00	0.01 ± 0.00	0.09 ± 0.00	#	#	0.06 ± 0.00	0.11 ± 0.00	0.03 ± 0.00	0.07 ± 0.00	0.05 ± 0.00
Rb1	8.26 ± 0.14	14.85 ± 0.04	19.9 ± 0.24	5.95 ± 0.01	2.85 ± 0.02	9.11 ± 0.05	12.60 ± 0.04	9.90 ± 0.14	12.03 ± 0.13	11.58 ± 0.09
Rc	4.08 ± 0.06	5.73 ± 0.11	8.63 ± 0.03	3.06 ± 0.02	2.16 ± 0.03	2.48 ± 0.08	4.03 ± 0.01	4.00 ± 0.04	4.17 ± 0.02	5.59 ± 0.19
Rb2	3.45 ± 0.01	4.57 ± 0.00	8.19 ± 0.01	2.87 ± 0.03	1.80 ± 0.01	2.20 ± 0.06	3.64 ± 0.08	3.61 ± 0.01	3.75 ± 0.06	5.23 ± 0.07
Rb3	0.77 ± 0.02	1.06 ± 0.01	1.69 ± 0.03	0.51 ± 0.03	0.36 ± 0.03	0.47 ± 0.02	0.83 ± 0.01	0.80 ± 0.05	0.79 ± 0.01	1.10 ± 0.02
Rd	0.73 ± 0.00	0.80 ± 0.00	1.74 ± 0.02	1.31 ± 0.02	0.93 ± 0.00	0.78 ± 0.01	1.90 ± 0.03	0.75 ± 0.01	0.78 ± 0.00	1.07 ± 0.04
Rh2	0.01 ± 0.00	0.04 ± 0.00	0.06 ± 0.00	0.02 ± 0.00	0.02 ± 0.00	0.01 ± 0.00	0.01 ± 0.00	0.01 ± 0.00	0.01 ± 0.00	0.01 ± 0.00
F2	0.01 ± 0.00	0.02 ± 0.00	0.01 ± 0.00	0.02 ± 0.00	0.03 ± 0.00	#	#	0.01 ± 0.00	0.01 ± 0.00	0.01 ± 0.00
Rg3	0.01 ± 0.00	0.01 ± 0.00	0.03 ± 0.00	0.01 ± 0.00	0.01 ± 0.00	0.01 ± 0.00	0.02 ± 0.00	0.01 ± 0.00	0.01 ± 0.00	0.02 ± 0.00
Ro	3.18 ± 0.02	2.09 ± 0.01	2.80 ± 0.02	3.40 ± 0.02	0.57 ± 0.01	2.81 ± 0.00	2.60 ± 0.03	1.08 ± 0.00	2.96 ± 0.00	1.06 ± 0.02
m-Rg1	0.71 ± 0.01	0.43 ± 0.01	0.41 ± 0.00	1.50 ± 0.01	0.39 ± 0.01	0.92 ± 0.01	0.68 ± 0.01	0.33 ± 0.01	0.50 ± 0.01	0.28 ± 0.01
m-Re	0.14 ± 0.00	0.29 ± 0.01	0.14 ± 0.00	0.38 ± 0.00	0.25 ± 0.01	0.07 ± 0.00	0.09 ± 0.00	0.11 ± 0.00	0.08 ± 0.00	0.10 ± 0.00
m-Rd	1.12 ± 0.01	1.25 ± 0.02	1.47 ± 0.01	3.75 ± 0.07	1.91 ± 0.01	0.98 ± 0.02	2.02 ± 0.02	0.88 ± 0.01	0.92 ± 0.01	1.24 ± 0.02
m-Rb1	11.54 ± 0.10	13.91 ± 0.08	13.16 ± 0.12	16.58 ± 0.04	6.09 ± 0.09	9.67 ± 0.05	11.28 ± 0.05	8.79 ± 0.29	9.85 ± 0.11	9.11 ± 0.01
m-Rc	5.40 ± 0.01	5.21 ± 0.01	5.12 ± 0.06	9.90 ± 0.25	4.66 ± 0.05	2.36 ± 0.02	3.40 ± 0.06	3.46 ± 0.05	3.31 ± 0.04	4.42 ± 0.07
m-Rb2	6.89 ± 0.05	5.79 ± 0.06	6.96 ± 0.07	12.87 ± 0.10	5.69 ± 0.04	2.69 ± 0.00	3.76 ± 0.02	4.05 ± 0.06	3.98 ± 0.01	5.65 ± 0.02
m-Rb3	1.26 ± 0.04	1.06 ± 0.03	1.14 ± 0.06	2.32 ± 0.02	0.91 ± 0.06	0.53 ± 0.03	0.74 ± 0.01	0.79 ± 0.04	0.67 ± 0.07	1.01 ± 0.01
Total	64.02 ± 0.22	81.57 ± 0.16	102.69 ± 0.91	78.88 ± 0.33	38.72 ± 0.15	57.08 ± 0.18	76.47 ± 0.14	58.28 ± 0.68	66.57 ± 0.25	70.99 ± 0.96
PPD/PPT	2.51 ± 0.00	2.16 ± 0.02	2.14 ± 0.01	3.63 ± 0.00	2.55 ± 0.01	1.36 ± 0.02	1.49 ± 0.01	1.84 ± 0.02	1.73 ± 0.01	1.93 ± 0.02
G/m-G	1.14 ± 0.00	1.71 ± 0.00	2.34 ± 0.01	0.55 ± 0.00	0.84 ± 0.00	1.95 ± 0.00	2.14 ± 0.00	1.92 ± 0.01	2.11 ± 0.02	2.01 ± 0.03

# not quantified. The total ginsenoside amount is the sum of all the quantified ginsenosides. The protopanaxadiol (PPD)-type amount and protopanaxatriol (PPT)-type amount are the sum of all the quantified PPD-type ginsenosides and PPT-type ginsenosides, respectively. G/m-G is the ratio of neutral ginsenoside amount to malonyl ginsenoside amount; the malonyl ginsenoside amount is the sum of seven quantified malonyl ginsenosides (m-Re, m-Rg1, m-Rb1, m-Rb2, m-Rb3, m-Rc, and m-Rd), and the neutral ginsenoside amount is the sum of corresponding neutral ginsenosides (Re, Rg1, Rb1, Rb2, Rb3, Rc, and Rd).

**Table 2:** Information of ten batches of ginseng samples.

NO	Sample	Age	NO	Sample	Age
1	Root, large and hard	13	6	Root, regular	12
2	Root, small and hard	13	7	Root, regular	12
3	Root, small and soft	13	8	Root, regular	12
4	Root, normal size and hard	8	9	Root, regular	12
5	Root, small and hard	8	10	Root, regular	12

## Supplementary Materials

The following are available online at https://www.mdpi.com/1420-3049/24/19/3491/s1, Inclusion articles lists.
