# Supplementary material for: Analysis of Ginsenoside Content (Panax ginseng) from Different Regions
Source: Molecules. 2019 Sep 26;24(19):3491. doi: 10.3390/molecules24193491 (PMC6803836; doi:10.3390/molecules24193491)
Supplement: Supplementary file 1 [file molecules-24-03491-s001.pdf]

Analysis of Ginsenoside Content (*Panax ginseng*) from Different Regions

Wei Chen [1,2,3], Prabhu Balan [2,3] and David G Popovich [1,*]

Articles inclusion about China grown ginseng

Cao, J. L., Li, Z. L., Fu, Q., Yan, D., Liao, Q. W., & Xiao, X. H. (2006). [The determination of contents of 8 ginsenosides in extraction of Panax ginseng by HPLC]. Zhong Yao Cai, 29(10), 1038-1040.

Chen, F., Luo, J., & Kong, L. (2013). Determination of 10 ginsenosides in Panax ginseng of different harvest times based on HPLC fingerprints and principal component analysis. Nat Prod Res, 27(9), 851-854. doi: 10.1080/14786419.2012.711758

Chen, R., Meng, F., Zhang, S., & Liu, Z. (2009). Effects of ultrahigh pressure extraction conditions on yields and antioxidant activity of ginsenoside from ginseng. Separation and Purification Technology, 66(2), 340-346. doi: https://doi.org/10.1016/j.seppur.2008.12.026

Chuang, W.-C., & Sheu, S.-J. (1994). Determination of ginsenosides in ginseng crude extracts by high-performance liquid chromatography. Journal of Chromatography A, 685(2), 243-251. doi: https://doi.org/10.1016/0021-9673(94)00724-1

Corthout, J., Naessens, T., Apers, S., & Vlietinck, A. J. (1999). Quantitative determination of ginsenosides from Panax ginseng roots and ginseng preparations by thin layer chromatography-densitometry. Journal of Pharmaceutical and Biomedical Analysis, 21(1), 187-192. doi: 10.1016/S0731-7085(99)00109-0

Gao, J., Zhai, Y., Wang, R., Wang, G., Wang, B., & Zhang, H. (2010). [Determination of eight kinds of ginsenosides in Shizhu ginseng]. Zhongguo Zhong Yao Za Zhi, 35(8), 989-991.

Li, W., & Fitzloff, J. F. (2002). HPLC analysis of ginsenosides in the roots of Asian ginseng (Panax ginseng) and North American ginseng (Panax quinquefolius) with in-line photodiode array and evaporative light scattering detection. Journal of Liquid Chromatography and Related Technologies, 25(1), 29-41. doi: 10.1081/JLC-100108537

Liu, Z., Li, Y., Li, X., Ruan, C.-C., Wang, L.-J., & Sun, G.-Z. (2012). The effects of dynamic changes of malonyl ginsenosides on evaluation and quality control of Panax ginseng C.A. Meyer. Journal of Pharmaceutical and Biomedical Analysis, 64-65, 56-63. doi: https://doi.org/10.1016/j.jpba.2012.02.005

MacCrehan, W. A., & White, C. M. (2013). Simplified ultrasonically- and microwave-assisted solvent extractions for the determination of ginsenosides in powdered Panax ginseng rhizomes

using liquid chromatography with UV absorbance or electrospray mass spectrometric detection. Anal Bioanal Chem, 405(13), 4511-4522. doi: 10.1007/s00216-013-6871-8

Qian, Z. M., Lu, J., Gao, Q. P., & Li, S. P. (2009). Rapid method for simultaneous determination of flavonoid, saponins and polyacetylenes in Folium Ginseng and Radix Ginseng by pressurized liquid extraction and high-performance liquid chromatography coupled with diode array detection and mass spectrometry. Journal of Chromatography A, 1216(18), 3825-3830. doi: https://doi.org/10.1016/j.chroma.2009.02.065

Shi, W., Wang, Y., Li, J., Zhang, H., & Ding, L. (2007). Investigation of ginsenosides in different parts and ages of Panax ginseng. Food Chemistry, 102(3), 664-668. doi: https://doi.org/10.1016/j.foodchem.2006.05.053

Wang, H. P., Zhang, Y. B., Yang, X. W., Zhao, D. Q., & Wang, Y. P. (2016). Rapid characterization of ginsenosides in the roots and rhizomes of Panax ginseng by UPLC-DAD-QTOF-MS/MS and simultaneous determination of 19 ginsenosides by HPLC-ESI-MS. J Ginseng Res, 40(4), 382-394. doi: 10.1016/j.jgr.2015.12.001

Wang, Y., You, J., Yu, Y., Qu, C., Zhang, H., Ding, L., . . . Li, X. (2008). Analysis of ginsenosides in Panax ginseng in high pressure microwave-assisted extraction. Food Chem, 110(1), 161-167. doi: 10.1016/j.foodchem.2008.01.028

Wu, C., Guan, Q., Wang, S., & Rong, Y. (2017). Simultaneous Determination of Multiple Ginsenosides in Panax ginseng Herbal Medicines with One Single Reference Standard. Pharmacogn Mag, 13(Suppl 1), S84-s89. doi: 10.4103/pm.pm_274_16

Wu, W., Sun, L., Zhang, Z., Guo, Y., & Liu, S. (2015). Profiling and multivariate statistical analysis of Panax ginseng based on ultra-high-performance liquid chromatography coupled with quadrupole-time-of-flight mass spectrometry. Journal of Pharmaceutical and Biomedical Analysis, 107, 141-150. doi: https://doi.org/10.1016/j.jpba.2014.12.030

Xie, Y. Y., Luo, D., Cheng, Y. J., Ma, J. F., Wang, Y. M., Liang, Q. L., & Luo, G. A. (2012). Steaming-induced chemical transformations and holistic quality assessment of red ginseng derived from panax ginseng by means of HPLC-ESI-MS/MS n-based multicomponent quantification fingerprint. Journal of Agricultural and Food Chemistry, 60(33), 8213-8224. doi: 10.1021/jf301116x

Xiu, Y., Li, X., Sun, X., Xiao, D., Miao, R., Zhao, H., & Liu, S. (2017). Simultaneous determination and difference evaluation of 14 ginsenosides in Panax ginseng roots cultivated in different areas and ages by high-performance liquid chromatography coupled with triple quadrupole mass spectrometer in the multiple reaction–monitoring mode combined with multivariate statistical analysis. Journal of Ginseng Research. doi: https://doi.org/10.1016/j.jgr.2017.12.001

Xu, X., Zheng, Y., Fu, S., Zhao, Y., Li, J., & Wang, L. (2011). [Determination of twelve ginsenosides in Panax ginseng by HPLC]. Zhongguo Zhong Yao Za Zhi, 36(11), 1463-1465.

Yamaguchi, H., Matsuura, H., Kasai, R., Tanaka, O., Kohda, H., Satake, M., . . . Goto, K. (1988). Analysis of Saponins of Wild Panax ginseng. Chemical and Pharmaceutical Bulletin, 36(10), 4177-4181. doi: 10.1248/cpb.36.4177

Yang, Y., Jiang, Y., & Zhang, L. (2017). Comparative analysis of ginsenosides in different growth ages and parts of Asian ginseng (Panax Ginseng C.A. Meyer) and American Ginseng (Panax Quinquefolius L.). Bangladesh Journal of Botany, 46(4), 1333-1340.

Ye, J., Gao, Y., Tian, S., Su, J., & Zhang, W. (2018). A novel and effective mode-switching triple quadrupole mass spectrometric approach for simultaneous quantification of fifteen ginsenosides in Panax ginseng. Phytomedicine, 44, 164-172. doi: 10.1016/j.phymed.2018.02.007

Zhang, H., Xu, S., Pang, S., Piao, X., & Wang, Y. (2018). Effect of seed size on seedling performance, yield and ginsenoside content of Panax ginseng. Seed Science and Technology, 46(2), 407-417. doi: 10.15258/sst.2018.46.2.22

Zhang, H., Xu, S., Piao, C., Zhao, X., Tian, Y., Cui, D., . . . Wang, Y. (2018). Post-planting performance, yield, and ginsenoside content of Panax ginseng in relation to initial seedling size. Industrial Crops and Products, 125, 24-32. doi: https://doi.org/10.1016/j.indcrop.2018.08.091

Zhang, L., Zhou, Q. L., & Yang, X. W. (2018). Determination of the transformation of ginsenosides in Ginseng Radix et Rhizoma during decoction with water using ultra-fast liquid chromatography coupled with tandem mass spectrometry. J Sep Sci, 41(5), 1039-1049. doi: 10.1002/jssc.201701228

Zhang, Y., Zhang, Y., Taha, A. A., Ying, Y., Li, X., Chen, X., & Ma, C. (2018). Subcritical water extraction of bioactive components from ginseng roots (Panax ginseng C.A. Mey). Industrial Crops and Products, 117, 118-127. doi: https://doi.org/10.1016/j.indcrop.2018.02.079

Zhang, Y. C., Li, G., Jiang, C., Yang, B., Yang, H. J., Xu, H. Y., & Huang, L. Q. (2014). Tissue-specific distribution of ginsenosides in different aged ginseng and antioxidant activity of ginseng leaf. Molecules, 19(11), 17381-17399. doi: 10.3390/molecules191117381

Zhen, G., Zhang, L., Du, Y. N., Yu, R. B., Liu, X. M., Cao, F. R., . . . He, H. (2015). De novo assembly and comparative analysis of root transcriptomes from different varieties of Panax ginseng C. A. Meyer grown in different environments. Science China Life Sciences, 58(11), 1099-1110. doi: 10.1007/s11427-015-4961-x

Zhou, Q.-L., Zhu, D.-N., Yang, X.-W., Xu, W., & Wang, Y.-P. (2018). Development and validation of a UFLC–MS/MS method for simultaneous quantification of sixty-six saponins and their six aglycones: Application to comparative analysis of red ginseng and white ginseng. Journal of Pharmaceutical and Biomedical Analysis, 159, 153-165. doi: https://doi.org/10.1016/j.jpba.2018.06.048

Zhou, S. S., Xu, J. D., Zhu, H., Shen, H., Xu, J., Mao, Q., . . . Yan, R. (2014). Simultaneous determination of original, degraded ginsenosides and aglycones by ultra high performance liquid chromatography coupled with quadrupole time-of-flight mass spectrometry for quantitative evaluation of Du-Shen-Tang, the decoction of ginseng. Molecules, 19(4), 4083-4104. doi: 10.3390/molecules19044083

Zhou, Z. H., & Zhang, G. D. (1988). [Analysis of ginseng. IV. HPLC determination of ginsenosides in Panax ginseng]. Yao Xue Xue Bao, 23(2), 137-141.

Articles inclusion about Korea grown ginseng

Gui, Y., & Ryu, G. H. (2013). The effect of extrusion conditions on the acidic polysaccharide, ginsenoside contents and antioxidant properties of extruded Korean red ginseng. J Ginseng Res, 37(2), 219-226. doi: 10.5142/jgr.2013.37.219

Ha, J., Shim, Y. S., Seo, D., Kim, K., Ito, M., & Nakagawa, H. (2013). Determination of 22 ginsenosides in ginseng products using ultra-high-performance liquid chromatography. J Chromatogr Sci, 51(4), 355-360. doi: 10.1093/chromsci/bms148

Hong, H. D., Sim, E. M., Kim, K., Rho, J., Rhee, Y. K., & Cho, C. W. (2009). Comparison of preparation methods for the quantification of ginsenosides in raw Korean ginseng. Food Science and Biotechnology, 18(2), 565-569.

Jiang, N. H., Lee, J. H., Jung, A. S., Jae, E. C., & Lee, K. T. (2008). Determination of ginsenosides content in Korean ginseng seeds and roots by high performance liquid chromatography. Food Science and Biotechnology, 17(2), 430-433.

Kim, D., Kim, M., Rana, G. S., & Han, J. (2018). Seasonal Variation and Possible Biosynthetic Pathway of Ginsenosides in Korean Ginseng Panax ginseng Meyer. Molecules, 23(7). doi: 10.3390/molecules23071824

Kim, S. N., Ha, Y. W., Shin, H., Son, S. H., Wu, S. J., & Kim, Y. S. (2007). Simultaneous quantification of 14 ginsenosides in Panax ginseng C.A. Meyer (Korean red ginseng) by HPLC-ELSD and its application to quality control. J Pharm Biomed Anal, 45(1), 164-170. doi: 10.1016/j.jpba.2007.05.001

Lee, G. J., Shin, B. K., Yu, Y. H., Ahn, J., Kwon, S. W., & Park, J. H. (2016). Systematic development of a group quantification method using evaporative light scattering detector for relative quantification of ginsenosides in ginseng products. J Pharm Biomed Anal, 128, 158-165. doi: 10.1016/j.jpba.2016.05.030

Lee, J. W., Choi, B. R., Kim, Y. C., Choi, D. J., Lee, Y. S., Kim, G. S., . . . Lee, D. Y. (2017). Comprehensive Profiling and Quantification of Ginsenosides in the Root, Stem, Leaf, and Berry of Panax ginseng by UPLC-QTOF/MS. Molecules, 22(12). doi: 10.3390/molecules22122147

Lee, J. W., Ji, S. H., Choi, B. R., Choi, D. J., Lee, Y. G., Kim, H. G., . . . Lee, D. Y. (2018). UPLC-QTOF/MS-based metabolomics applied for the quality evaluation of four processed panax ginseng products. Molecules, 23(8). doi: 10.3390/molecules23082062

Li, X., Nam, K., & Choi, J. (2009). Difference of the ginsenosides contents according to the planting location in Panax ginseng C. A. Meyer. Korean Journal of Crop Science / Hanguk Jakmul Hakhoe Chi, 54(2), 159-164.

Li, X. G., Yan, Y. Z., Jin, X. J., Kim, Y. K., Uddin, M. R., Kim, Y. B., . . . Park, S. U. (2012). Ginsenoside content in the leaves and roots of Panax ginseng at different ages. Life Science Journal, 9(4), 679-683.

Park, H. W., In, G., Han, S. T., Lee, M. W., Kim, S. Y., Kim, K. T., . . . Chang, I. M. (2013). Simultaneous determination of 30 ginsenosides in Panax ginseng preparations using ultra performance liquid chromatography. J Ginseng Res, 37(4), 457-467. doi: 10.5142/jgr.2013.37.457

Park, S. U., Ahn, D. J., Jeon, H. J., Kwon, T. R., Lim, H. S., Choi, B. S., . . . Bae, H. (2012). Increase in the Contents of Ginsenosides in Raw Ginseng Roots in Response to Exposure to 450 and 470 nm Light from Light-Emitting Diodes. J Ginseng Res, 36(2), 198-204. doi: 10.5142/jgr.2012.36.2.198

Sun, B.-S., Gu, L.-J., Fang, Z.-M., Wang, C.-y., Wang, Z., Lee, M.-R., . . . Sung, C.-K. (2009). Simultaneous quantification of 19 ginsenosides in black ginseng developed from Panax ginseng by HPLC–ELSD. Journal of Pharmaceutical and Biomedical Analysis, 50(1), 15-22. doi: https://doi.org/10.1016/j.jpba.2009.03.025

Sun, B. S., Gu, L. J., Fang, Z. M., Wang, C. Y., Wang, Z., & Sung, C. K. (2009). Determination of 11 ginsenosides in black ginseng developed from Panax ginseng by high performance liquid chromatography. Food Science and Biotechnology, 18(2), 561-564.

Wang, H. P., Zhang, Y. B., Yang, X. W., Zhao, D. Q., & Wang, Y. P. (2016). Rapid characterization of ginsenosides in the roots and rhizomes of Panax ginseng by UPLC-DAD-QTOF-MS/MS and simultaneous determination of 19 ginsenosides by HPLC-ESI-MS. J Ginseng Res, 40(4), 382-394. doi: 10.1016/j.jgr.2015.12.001

Yang, B. W., Lee, J. B., Lee, J. M., Jo, M. S., Byun, J. K., Kim, H. C., & Ko, S. K. (2019). The Comparison of Seasonal Ginsenoside Composition Contents in Korean Wild Simulated Ginseng (Panax ginseng) which were Cultivated in Different Areas and Various Ages. Natural Product Sciences, 25(1), 1. doi: 10.20307/nps.2019.25.1.1

Articles inclusion about New Zealand grown ginseng

Follett, J. M., Proctor, J. T., Walton, E. F., Boldingh, H. L., McNamare, C., & Douhlas, J. A. (2004). Carbohydrate and Ginsenoside Changes in Ginseng Roots Grown in the Bay of Plenty New Zealand. J Ginseng Res, 28(4), 165-172.
